# Nucleic acids delivery methods for genome editing in zygotes and embryos: the old, the new, and the old-new

**DOI:** 10.1186/s13062-016-0115-8

**Published:** 2016-03-31

**Authors:** Masahiro Sato, Masato Ohtsuka, Satoshi Watanabe, Channabasavaiah B. Gurumurthy

**Affiliations:** Section of Gene Expression Regulation, Frontier Science Research Center, Kagoshima University, 8-35-1 Sakuragaoka, Kagoshima, Kagoshima, 890-8544 Japan; Division of Basic Molecular Science and Molecular Medicine, School of Medicine, Tokai University, Kanagawa, 259 1193 Japan; Animal Genome Research Unit, Division of Animal Science, National Institute of Agrobiological Sciences, Ibaraki, 305-8602 Japan; Developmental Neuroscience, Munroe-Meyer Institute, University of Nebraska Medical Center, Omaha, NE 68198 USA; Mouse Genome Engineering Core Facility, University of Nebraska Medical Center, Omaha, NE 68198 USA

**Keywords:** Genome editing, CRISPR/Cas, Pronuclaer injection, Transgenic, Nucleic acids delivery, Mcroinjection

## Abstract

In the recent years, sequence-specific nucleases such as ZFNs, TALENs, and CRISPR/Cas9 have revolutionzed the fields of animal genome editing and transgenesis. However, these new techniques require microinjection to deliver nucleic acids into embryos to generate gene-modified animals. Microinjection is a delicate procedure that requires sophisticated equipment and highly trained and experienced technicians. Though over a dozen alternate approaches for nucleic acid delivery into embryos were attempted during the pre-CRISPR era, none of them became routinely used as microinjection. The addition of CRISPR/Cas9 to the genome editing toolbox has propelled the search for novel delivery approaches that can obviate the need for microinjection. Indeed, some groups have recently developed electroporation-based methods that have the potential to radically change animal transgenesis. This review provides an overview of the old and new delivery methods, and discusses various strategies that were attempted during the last three decades. In addition, several of the methods are *re*-*evaluated* with respect to their suitability to deliver genome editing components, particularly CRISPR/Cas9, to embryos.

**Reviewers:** Drs. Eugene Koonin and Haruhiko Siomi.

## Background

Targeted gene inactivation via homologous recombination (HR) using embryonic stem (ES) cells has been a powerful tool to evaluate the function of a gene of interest (GOI) in vivo [1]. However, the use of this technique has been hampered by several factors, including: the low efficiency of targeting in ES cells; the need for time-consuming and labor-intensive screening of ES clones; the maintenance of clones under undifferentiated states; chimeric mouse production; and breeding chimeras to obtain germ-line transmission of the mutation.

**Table 1:** Nucleic acids delivery methods in animal transgenesis and genome editing

Category	Method	Remarks	Germ line transmission potential
Ex vivo approaches	Pronuclear injection	The most commonly used method followed by thousands of labs for over 3 decades	High
	Viral Vectors	A few labs used. Limited success.	High when lentiviral vectrs are used
	Receptor-mediated uptake	Only one report [28].	Not proven
	In vitro electroporation	Novel approach: also proven using CRISPR system.	High
	Liposomal transfection	Very few labs used. Limited success.	Not proven
	Blastocyst microinjection	Only one report [32]: may be suitable for expression analysis in embryonic tissues.	Not proven
	Sperm-mediated gene transfer (SMGT)	Very few labs have attempted. Limited success.	Low
	Intracytoplasmic sperm injection-mediated gene transfer (ICSI-MGT)	Very few labs have attempted. Limited success.	Low
In vivo delivery to pre-implantation embryos, fetuses and ovarian tissues	GONAD	Only one report [50]. This method completely eliminates the need for isolation, microinjection and transfer of embryos to recipient mice. Only one recent so far, yet to be tested in other labs.	Not proven yet, but highly likely
	Trans-placental gene delivery to fetuses	Very few labs have attempted. Limited success.	Very low
	Delivery to fetal tissues in utero	Very few labs have attempted. Limited success.	Very low
	In vivo delivery to ovarian tissues	Very few labs have attempted. Limited success.	Low
In vivo delivery to male gonadal tissues	Testis-mediated gene transfer (TMGT)	Several labs have attempted. Limited success.	Possible, may need to screen many offspring from the treated males
	Seminiferous tubule-mediated gene delivery	A few labs have attempted. Limited success.	
	Gene delivery via vas deferens	Very few labs have attempted. Limited success.	
	Nucleic acids delivery to the cauda epididymis	Very few labs have attempted. Limited success.	

Recently, genome editing using sequence-specific nucleases has emerged as a new tool for achieving targeted mutations in GOI in a wide variety of cell types and organisms [2–4]. To date, three different systems—zinc-finger nuclease (ZFN), transcription activator-like effector nuclease (TALEN), and clustered regulatory-interspaced short palindromic repeats (CRISPR/Cas: CRISPR-associated 9 and CRISPR/Cas9—use sequence-specific nucleases for efficient and precise genetic modifications. They induce targeted double-strand breaks (DSBs), which stimulate cellular DNA repair mechanisms such as non-homologous end joining (NHEJ) and homology-directed repair (HDR) [5, 6]. Owing to the ease of its design and flexibility, the CRISPR/Cas9 system is now widely used for producing genetically modified animals, including the mouse [7].

Animal genome engineering labs have readily adapted these newer technologies. Nevertheless, the delivery of these nucleic-acid tools to embryos still relies on the three-decades-old transgenic procedures of embryo isolation, micro-injection, and transfer of embryos to recipients. These traditional transgenic techniques require expensive micromanipulator systems and highly skilled technical personnel to perform the procedure. Developing newer methods can obviate the need for expensive equipment and highly skilled personnel.

Over the past three decades, several methods have been described for delivering transgenic DNA through various modes, such as pre-implantation embryos/fetuses, ovarian cells (including oocytes), and testicular cells (including sperm), to achieve a desired genetic change in the offspring. While such methods have been partially successful in reaching the embryos in situ, additional strategies are necessary to efficiently deliver genome editing components. The latest additions to these approaches include electroporation-based methods that bypass microinjection. One of the most recent methods developed by us also bypasses the isolation and transfer of embryos into recipient animals.

Here, we review over a dozen different gene delivery approaches that were tried but did not become commonly used approaches. In light of recent advances in CRISPR/Cas9 technology, which requires the development of simpler and better electroporation-based approaches, the large array of gene delivery methods that had been previously tried could be re-visited and fine-tuned to establish their suitability for use with CRISPR gene editing tools. These delivery approaches are discussed below (sections Ex vivo delivery to pre-implantation embryos and to sperm, In vivo delivery to pre-implantation embryos, fetuses, and ovarian tissues & In vivo delivery to male gonadal tissues) under three broad sections: Ex vivo delivery to pre-implantation embryos and sperm; In vivo delivery to pre-implantation embryos, fetuses and ovarian tissues; and In vivo delivery to male gonadal tissues. Various nucleic acids delivery methods discussed in this review are listed in Table 1.

## Ex vivo delivery to pre-implantation embryos and to sperm

### Pronuclear injection (PI)

**Fig. 1:**
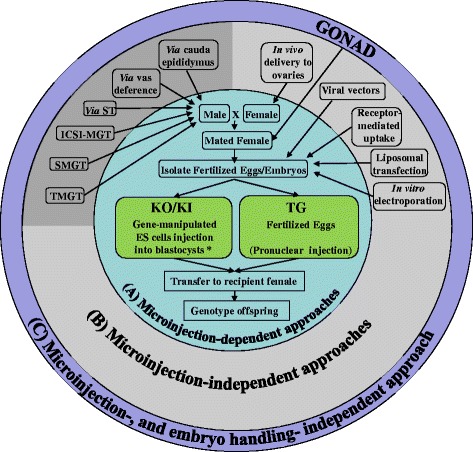
Schematic showing various Knoc-kout/Knock-In (KO/KI) and Transgenic (TG) mice generation approaches reviewed in the article. Only the methods that have potential for generating germ-line transmitted offspring are shown (see text for additional methods that may not have germ-line transmission potential). (*A*) The classical methods that require microinjection are shown in inner circle, [*; the CRISPR system can generate KO/KI models directly through PI and therefore can by-pass the use of ES cells]. (*B*) The approaches that do not require microinjection are listed in the middle circle (those in the dark gray shaded area do not require ex vivo handling of embryos). (*C*) The GONAD method that does not require both microinjection- and ex vivo handling- of embryos, is listed in the outer circle

Microinjection of purified DNA, commonly known as pronuclear injenction (PI), into the pronuclei of zygotes to produce Tg mice was first demonstrated by Gordon et al., 1980 [8] (schematically shown in Fig. 1a). Since then, the PI method has been perfected and used in many labs; they are primarily used to introduce a transgene harboring the GOI into the genome to generate Tg animals. PI technique involves the micro-injection of purified nucleic-acids into fertilized eggs (or pronuclei), and until recently, the PI approach was thought to be impossible for generating knock-out (KO) and knock-in (KI) animals. It was demonstrated for the first time that KO models could be generated by direct PI approach by injecting ZFNs; these were first introduced in 2009 for genome editing [9]. Other, newer techniques such as PITT (Pronuclear Injection-based Trageted Transgenesis) [10], TALENs, and CRISPR/Cas9 systems have employed PI to generate KO/KI animals. Though PI is a fairly simple and rapid method for the creation of KO animals compared to traditional, targeted gene modification systems using ES cells, PI requires the use of expensive micromanipulation equipment and skilled peronnel to operate the equipment.

### Delivery through retrovirus, lentivirus, and adenovirus infections

Although less common, viral delivery systems are used as delivery agents for Tg DNA by infecting pre-implantation embryos with viral vectors, such as a retrovirus [11–13], lentivirus [14] or adenovirus [15, 16]. A seminal experiment of viral vector delivery was reported by Tsukui et al., 1996, who used a replication-defective adenovirus vector carrying *lacZ* to zona pellucida (ZP)-free mouse zygotes [16] (schematic shown in Fig. 1b). The authors also demonstrated the presence of transgenes in the genomes of the live offspring. Many subsequent attempts using retroviral and lentiviral vectors (not cited here) suggest that transgenesis via the viral infection of early embryos is effective approach to create Tg animals.

The two major advantages of an adenoviral system that are particularly suited for delivery of CRISPR tools are that i) adenoviruses, unlike retro-/lenti-viruses, do not need to be integrated into the genome [17]; and ii) adenoviruses generally have higher infection efficiency than retro-/lenti-viruses. Adenoviral systems have been reported to be superior to lentiviral systems for accurate genome editing with engineered nucleases in human cells [18]. Adeno-associated virus (AAV) is an alternative choice for delivery of CRISPR/Cas9 components. AAV is a versatile and safe vector system and is used in therapeutic applications but one of the limitation of AAV is its small cargo size that it can accommodate (around 4.5 kb in length). While wildtype Cas9 is not suitable for packaging into AAV, a recently identified Cas9 from *Staphylococcus aureus* (saCas9) could be packaged into AAV because of its shorter size [19]. A major, inconvenient step required for using viral vectors is the preparation and concentration of viral particles prior to infection, both time-consuming and labor-intensive.

### In vitro electroporation

Grabarek et al., 2002 demonstrated for the first time that nucleic acids can be efficiently delivered to isolated oocytes and zygotes by electroporation [20]. One disadvantage of this method, however, is that the ZP must be removed, and this often hampers the effective uptake of DNA. Also, ZP-free embryos cannot survive in vivo, since they tend to be trapped by the inner surface of the oviductal wall due to their adhesiveness, leading to reduced pregnancy rates among the embryo-recipient animals [21, 22]. To overcome this issue, in vitro cultivation of treated zygotes was considered to develop further into blastocysts and for subsequent transfer to recipient females. Grabarek et al., 2002 tried an alternative strategy to weaken the ZP by a brief treatment with acidic Tyrode’s solution, which then allowed the enhanced uptake of exogenous DNA and protected the embryos from electroporation damage [20]. Many groups subsequently tried the zona weakening strategy and succeeded in electroporation-mediated delivery of nucleic acids to early embryos [23, 24].

The first successful genome editing in isolated rat zygotes through electroporation was reported in 2014 by Kaneko et al., [25], who used in vitro electroporation-mediated gene delivery (shown in Fig. 1c), and reported up to 73 % efficiency with this approach in genome editing by delivering TALENs or CRISPR/Cas9. A new electroporation apparatus, called NEPA 21 (NEPA GENE), was used in this report: the apparatus generates a set of electric pulses that are mainly comprised of poring pulses and transfer pulses. The poring pulse creates a transient poration of the cell membrane, and the tranfer pulse elicits the transfer of exogenous DNA into the cell or embryo via the pores (http://www.nepagene.jp/index2.html). According to the authors, electroporation with the defined set of pulses resulted in efficient nucleic acid-delivery, and resulted in increased embryo viability. Using this method, a large number of embryos (~100) can be handled at once, as opposed to micro-injection performed on one embryo at a time, and this method does not require ZP weakening. The success of the Kaneko's group may be attributed to the capabilities of NEPA 21 itself, which permits the rapid transfer of exogenous DNA inside the cells and embryos, while reducing cellular damages caused by electrical heating. Soon after this report, two other groups used in vitro electroporation for successful genome editing in pre-implantation embryos [26, 27], although they employed electroporators that were different from NEPA 21. Even though “in vitro electroporation in isolated embryos” made a significant advance in delivering nucleic acids to embryos, this method still requires that embryos be isolated, handled outside the animal, and transferred back to a different set of females.

### Nucleic acid delivery via receptor-mediated uptake

This method relies on the uptake of exogenous DNA via receptors expressed on the surface of cells and embryos. The “receptor-mediated gene transfer” was first demonstrated by Ivanova et al., 1999 [28]. Mouse and rabbit pre-implantation embryos were incubated with intact ZPs for 3 h with plasmid DNA- containing insulin as an internalizable ligand, and it was observed that the constructs penetrated the ZP and accumulated in the peri-nuclear space of the blastomere. The exogenous DNA was also confirmed as integrated into the genome of fetuses and newborns that were derived from the treated embryos. According to the authors, the construct containing DNA and insulin does not bind to the ZP, but penetrates inside to accumulate in the peri-nuclear space of the embryos at various stages, from zygote to morula. Although this approach has not become popular, it suggests that delivery via receptor-mediated uptake can be used for the creation of genome-edited animals without resorting to PI- or electroporation-based approaches.

### Liposomal transfection

The ZP-denuded preimplantation embryos were successfully transfected with plasmid DNAs, similar to liposome-mediated transfection in cultured cells [29, 30] (shown in Fig. 1d). However, the transfection efficiency was relatively low, attributed to ZP acting as a barrier and preventing transfection. Furthermore, Tg founders were obtained by transferring the liposomally transfected embryos to the reproductive tract of recipients, although its overall efficiency was only 1.27 % [30]. Although these methods did not result in germ line transmission of transgenes, there are some advantages of liposome delivery: i) it does not require sophisticated equipment (such as micromanipulator or electroporator); ii) it is less toxic than electroporation; and iii) a large number of embryos (~100) can be processed simultaneously. On the other hand, this method suffers similar shortcomings as others, as shown for in vitro electroporation, because it requires ZP-denuded (not ZP-intact) embryos, the use of which leads to reduced pregnancy rates in recipient animals. Notably, Joo et al., 2014, recently developed a hydrophilic and Cy5.5-labeled organic compound called VisuFect, and it was demonstrated that VisuFect that had been conjugated with poly(A) oligo (referred to as ‘VFA’) successfully penetrated through the ZP of the fertilized eggs of various species, including those of pigs, zebrafish, fruit flies, and mice [31]. This suggests that VisuFect could be used to deliver genome-editing nucleic acids to ZP-intact embryos to generate genetically modified animals without affecting pregnancy rates in the recipient animals.

### Blastocyst microinjection

This method is intended to deliver nucleic acids directly to cells in the inner cell mass (ICM), a precursor of the fetus, in a blastocyst. This approach was first demonstrated by Jaenisch and Minz, 1974, who microinjected simian virus 40 (SV40) viral DNA into the cavity (called the ‘blastocoel’) of the blastocyst [32] (shown in Fig. 1e); this resulted in successful infection of a portion of cells in both the trophectodermal layer and the inner cell mass (ICM). After surgical transfer to recipient females, approximately 40 % of the founders born possessed the exogenous DNA in the genomes of some organs; however, the germ line transmission of the genetic mutation was not demonstrated in this report. This method is not followed routinely for the obvious reason that not all cells will take the foreign nucleic acids, and leads to a high degree of mosaicim and the need to extensively screen several independent offspring, even if germ line transmission occurs. This method requires a micromanipulator system for micro-injection, and also requires the transfer of treated blastocysts into the uteri of recipient females.

### Sperm-mediated gene transfer (SMGT)

Lavitrano et al., 1989 [33] demonstrated for the first time that brief incubation of spermatozoa, isolated from cauda epididymides and with circular plasmids, resulted in the adhering of spermatozoa to DNA (shown in Fig. 1f). When these DNA-bound spermatozoa were used in in vitro fertilization (IVF) with oocytes, the resulting zygotes harbored the exogenous DNA in their genome. This method was called “sperm-mediated gene transfer” (SMGT). Because of its simplicity and it did not require expensive micromanipulator systems, this technology was regarded as a more convenient tool for the production of Tg animals than the PI-based transgenesis. This technique was later tried in many laboratories, but results have been mixed [34, 35]; so far, only a limited number of laboratories have reported successful data using SMGT [36–39]. If the delivery of CRISPR/Cas9 genome-editing components is attempted through SMGT, sperm might need to be incubated in a medium containing the plasmid DNA that encodes Cas9 and single-guide (sg)RNA or mRNA for Cas9 and sgRNA. As mentioned above, SMGT is a very simple method: if successful genome editing using this system is successfully developed, it can be used routinely.

### Intracytoplasmic sperm injection-mediated gene transfer (ICSI-MGT)

It is possible to generate live offspring by microinjecting inactive or dead spermatozoa into the cytoplasm of a normal oocyte. This was first demonstrated by Lin, 1969 [40], and is now called intracytoplasmic sperm injection (ICSI). Perry et al., 1999 [41], demonstrated that Tg mice can be produced when dead spermatozoa incubated with exogenous DNA are microinjected into normal eggs (shown in Fig. 1g). Since this first demonstation, this transgenesis technology has been tried by others and called by different names such as ICSI-MGT or TransICSI [38, 42, 43]. This technology is particularly useful for the introduction of larger transgenes derived from bacterial artificial chromosomes (BAC) into the animal genome [44, 45].

## In vivo delivery to pre-implantation embryos, fetuses, and ovarian tissues

The approaches mentioned in Section "Ex vivo delivery to pre-implantation embryos and to sperm" involve the isolation of zygotes, oocytes, sperm, and embryos, their ex vivo treatment to deliver nucleic acids, and subsequent transfer to recipient females for further development. In this section, we summarize the methods that do not need isolation and ex vivo handling steps, but that can deliver the nucleic acid directly in situ.

### GONAD

**Fig. 2:**
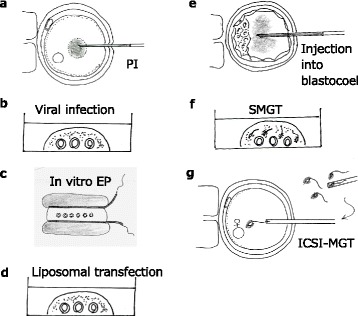
Ex vivo delivery methods to pre-implantation embryos and sperm

Delivery of liposomally encapsulated DNA directly into the oviductal lumen was first reported by Esponda’s group [46, 47] (shown in Fig. 2h). In 2005, we attempted to deliver plasmid DNA to oviductal epithelium by in vivo instillation into oviductal lumen (ampulla) at Day 0.4 of pregnancy (corresponding to the zygote stage), and by subsequent in vivo electroporation towards the entire ovuduct using tweezer-type electrodes [48]. The plasmid DNA was delivered in up to 43 % of the oviductal epithelium; we named the method gene transfer to oviductal epithelium (GTOVE). This approach was initially meant to deliver DNA to the zygotes residing at the ampulla, but we failed to accomplish this goal. In all likelihood, the presence of cumulus cells surrounding the oocytes might have acted as a barrier hindering DNA uptake by the zygotes. We then tried the technique at day 1.6 (~14:00 h on the day of procedure) of pregnancy, believing that the cumulus cells might be detached from the embryos at this stage, and that gene delivery to the embryos might therefore occur. This, indeed, turned out to be the case: we observed that fluorescent 4–8 cell embryos that had been isolated from the oviducts dissected 1 day after GTOVE using EGFP plasmid [49]. Unfortunately, chromosomal integration of the exogenous DNA was not achieved in offspring analyzed at the mid-gestational stage. The transient nature of this transgene expression then prompted us to test the CRISPR/Cas9 system, which causes indel mutations at the target locus in 2-cell embryos through a hit-and-run mechanism, with the CRISPR components only having to cleave the genomic DNA without needing to integrate into the genome. Further, we recently demonstrated that the instillation of Cas9 mRNA and sgRNA into the oviductal lumen at day 1.6 of gestation and subsequent in vivo electroporation using a T820 electroporator (BTX, San Diego, CA, USA) resulted in the generation of embryos and fetuses with mutations at the target locus [50]. The efficiency of mutations among the fetuses isolated was relatively low: of the 25 fetuses obtained, only 7 of them (28 %) had mutations [50]. Nevertheless, these data clearly indicate that in vivo genome editing by direct delivery of genome editing components to preimplantation embryos is possible. Accordingly, we re-named this technology as *G*enome-editing via *O*viductal *N*ucleic *A*cids *D*elivery (GONAD) [50].

GONAD technology is the simplest and most convenient method among all the existing gene delivery systems targeted to pre-implantation embryos. It does not require many complex steps for animal transgenesis, such as: (i) isolation of embryos; (ii) ex vivo handling and culturing of embryos; (iii) manipulation of embryos such as microinjection; or (iv) microsurgery to transfer embryos to recipient females. GONAD can be used for genome editing in other species where ex vivo handling of isolated zygotes is considered difficult (for example, pigs and calves). The original GONAD was performed at the 2-cell stage, where it often caused mosaic patterns of gene expression [50]. One of the obvious reasons for such mosaicism could be that the components introduced by GONAD (such as EGFP or Cas9 mRNA and sgRNA) might have differed between the two blastomeres at the 2-cell embryo stage, causing differential genome editing activity in the offspring of each blastomere. To prevent such mosaicism in this context, it may be required to use GONAD at the zygote (1-cell) stage (Day 0.4 [~10:00 h] of gestation).

### Trans-placental gene delivery to fetuses

Tsukamoto et al., 1995 demonstrated for the first time that exogenous DNA (encapsulated by liposomes), administrated through the tail vein of pregnant females, could be transferred to fetuses via the placenta [51] (shown in Fig. 2k). Some of the fetuses exhibited blue deposits (β-galactosidase-catalayzed reaction products when X-Gal was used as a substrate) throughout the body, showing successful gene delivery and expression. Since then, several laboratories, including ours, have succeeded in delivering nucleic acids to post-implantation embryos and fetuses [52, 53]. O’Shea et al. [53] systemically administered short hairpin (sh) RNAs to mothers during the early post-implantation stage of gestation, and observed gene knockdown and defects that phenocopied the null embryo. The authors concluded that the systemic delivery of shRNAs is a feasible approach to gene silencing in the embryo. To date, however, there are no reports of successful germ-line transmission of the transferred gene using these approaches. Although a thorough analysis of the distribution of introduced DNA among fetal tissues has not been performed, delivery to organs in the circulatory system, such as the heart and blood vessels, may be readily achieved, since the exogenous DNA is delivered via the placenta through fetal vessels. It is likely that if guide RNA and Cas9 are expressed under a tissue-specific promoter, it could be possible to generate animals with genetic mutations in the target organs or tissues.

### Delivery to fetal tissues in utero

Direct delivery to post-implantation embryos and fetuses is another approach for achieving expression of the gene in specific target tissues. For example, Tabata and Nakajima, 2001 reported an efficient in utero gene transfer system to the developing mouse brain using electroporation [54]. The method is performed by surgical exposure of the uterus carrying the fetuses, direct injection of the exogenous DNA into fetal tissues, and in vivo electroporation at the injected sites (shown in Fig. 2i). The in utero delivery approach has been successfully achieved for various tissues, including those involving the skin [55], lungs [56], and brain [57]. However, examples of targeting to germ cells at the embryonic stage do not exist because the germ cell tissue and lineage is very poorly developed at this stage. Even though such targeted gene delivery approaches to fetal tissues are useful for studies intended for fetal genome editing, they cannot be used for achieving germ-line transmittable mutations.

### In vivo delivery to ovarian tissues

There are a few examples of delivering exogenous DNA to ovarian cells either through viral vectors (Gordon 2001 [58]) or liposomal delivery systems (Shimizu et al., 2004 [59]). Gordon 2001 [58] reported infecting ovarian cells and oocytes by direct injection of adenoviral vectors into the medulla of an ovary. However, no transduction of oocytes was observed, despite administering high doses of viral particles. Shimizu et al., 2004 [59] injected liposomally encapsulated plasmid DNA in to the medullae of porcine ovaries. The plasmid encoded porcine growth differentiation factor-9 (GDF-9), a growth-stimulating factor belonging to the TGF-β family of proteins is expressed specifically in immature oocytes. The injection resulted in an increased growth of immature follicles and led to an increase in the number of secondary and tertiary follicles [59]. The authors attributed this phenomenon to an increased production of GDF9 in the transfected ovaries; however, they did not report the detailed localization of gene products in the ovary expressed from the introduced DNA.

We explored another strategy, delivering plasmid DNA to ovarian cells (including oocytes) by intraovarian injection, followed by in vivo electroporation [60] (shown in Fig. 2j). In these experiments, we demonstrated that about 20 μl solution can be injected into an ovary at the weaning age. Histological examination of the ovaries indicated that 8–60 % of the follicles exhibited reporter (lacZ)-derived blue deposits. Some oocytes that were surrounded by one or two follicular layers (but not those surrounded by multi-layered follicles) were also positive for staining for lacZ activity. We noted exogenous gene expression up to about 1 week after the procedure [60]. Interestingly, Yang et al., 2007 used a similar approach to ours and reported that the DNA injected into the ovary was incorporated into the oocytes, and that the oocytes that matured were ovulated after hormonal stimulation [61]. They further observed that the ovulated oocytes resulted in transmission of exogenous DNA to the F0 offspring derived from them. This illustrates the possibility of Tg mouse production with relative ease by ovarian delivery, although it remains unclear how the introduced exogenous DNA is incorporated into the oocytes within an ovary, or how it is transmitted to the F0 offspring via fertilization. It will be interesting to test whether direct delivery of genome-editing components to ovaries in situ can edit the genome of immature oocytes, and whether they develop further into oocytes capable of generating genetically modified animals.

## In vivo delivery to male gonadal tissues

### Testis-mediated gene transfer (TMGT)

**Fig. 3:**
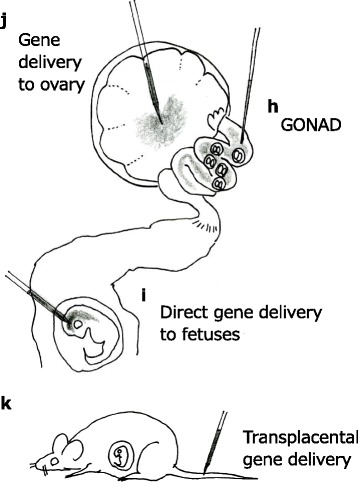
In vivo delivery to pre-implantation embryos, fetuses and ovarian tissues

We demonstrated for the first time that the plasmid DNA injected into the interstitial space of testis was detected in the cauda epididymis, up to 1 week after DNA injection [62]. This suggests that testicular spermatozoa can receive exogenous DNA and can then mature and be transferred to the cauda epididymis (shown in Fig. 3l). Others have also reported injecting plasmid DNAs to testicular spermatozoa using a glass pipette or 30-G needle [63–65]. Blanchard and Boekelheide, 1997 delivered an adenovirus vector to the interstitial compartment of adult rat testes by intratesticular injection and found transgene expression in Leydig cells [65]. Muramatsu, 2000 demonstrated that the intratesticular injection of DNA and subsequent in vivo electroporation resulted in the delivery of DNA to testicular spermatozoa as well as to interstitial cells [66].

**Fig. 4:**
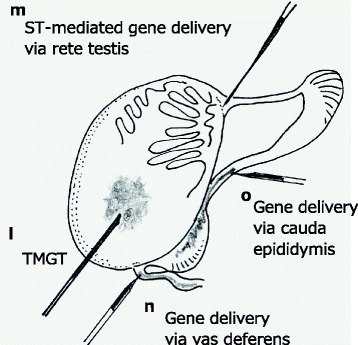
In vivo delivery to male gonadal tissues

When males injected with exogenous DNA using the intratesticular injection methods were mated to superovulated females, transmission and expression of the DNA was verified at the blastocysts and fetuses [67–71]. This method, based on gene delivery to offspring via testis and subsequent natural fertilization by mating with females, is termed “testis-mediated gene transfer” (TMGT) [70], and serves as a more conventional and simpler approach for the production of Tg mice, with several laboratories having successfully used this technology [72–74]. However, the technology has been debated for its suitability as a commonly-usable method for generating transgenic models because, first, the transgene insertion is not guaranteed in every sperm. To ensure the success of each transgenic project, a vast number of offspring needs to be screened for the presence of transgene and analyzed for transgene expression before establishing a breeder colony. Second, exogenous DNA introduced by the TMGT is mainly limited to the interstitial space of each testis. It should be noted that although there are some reports showing that exogenous DNA delivered to seminiferous tubules (ST) can yield Tg offspring [73], it is rare that DNA will enter into the lumen of the ST containing spermatogonial (immature sperm) stem cells that later develop into mature sperm cells [75]. Third, in this method, it is observed that the DNA transmitted to the offspring occasionally gets fragmented, making it unsuitable for Tg animal production [76]. Lastly, the copy number of plasmid DNA transmitted to the offspring after mating TMGT-treated males with normal females was below 1 copy per cell [70], and it can therefore be very difficult to detect the transgene by genomic Southern blotting. Although TMGT has such problems, it still holds some promise for use as a tool for KO animal production through sequence-specific nucleases (ZFNs, TALENs or CRISPR/Cas9) that cause mutations through a hit-and-run mechanism and do not require chromosomal integration of transgenes into the sperm genome.

### ST-mediated gene delivery

ST-mediated gene delivery is usually performed by the direct injection of DNA into the lumen of STs exposed above the testicular capsule [65, 77, 78], or of STs via rete testis, by a micromanipulator-controlled glass micropipette [79] (shown in Fig. 3m). Through ST-mediated delivery, the likelihood of delivering nucleic acids to spermatogonia and other differentiated spermatogenic cells, such as spermatid and spermatocytes, is higher compared to that achieved by TMGT. In fact, successful delivery to spermatogenic cells was achieved by many groups [65, 77, 78, 80], although gene transmission after the natural mating of treated males with females was not sufficiently examined. For example, while Yamazaki et al., 2000 [78] provided no evidence for gene transmission to the offspring, Celebi et al., 2000 [80], showed that the transgene was transmitted to the offspring, but nevertheless remained episomal, detected in the tail of young animals but not adults. It was therefore concluded that the plasmid was lost during the numerous germ cell divisions. These observations and reports suggest that ST-mediated gene delivery can also be a tool to create genome-edited KO animals as an alternative to the widely used zygote injection-based genome editing.

### Gene delivery via vas deferens

Huguet and Esponda, 1998, first demonstrated that injection of a liposome-DNA complex into the lumen of the vas deferens, using a glass micropipette, could deliver the DNA to mature spermatozoa in the lumen of the proximal region of the vas deferens [81] (shown in Fig. 3n). The same group also showed that 13.3 % of the epithelial cells of the vas deferens received the DNA by this method [82]. However, gene transmission to offspring via mating with females was not demonstrated by this method. This approach seems to be potentially useful because it can directly deliver nucleic acids to mature spermatozoa, which would be ejaculated during mating soon after. Additionally, gene delivery via the vas deferens is technically simpler and more convenient than ST-mediated gene delivery, since the vas deferens lumens are relatively larger than those of STs.

### Nucleic acids delivery to the cauda epididymis

The cauda epididymis is the site where sperm undergo final maturation and also is the site of sperm storage. Using a glass microcapillary needle, Esponda and Carballada, 2009 injected 1–2 μl of the DNA/liposome mixture into the lumen of the distal region of mouse cauda epididymis and detected fluorescence in the nucleus and cytoplasm of epithelial cells [83] (shown in Fig. 3o). Kirby et al., 2004 also reported a similar strategy where they instilled DNA into the lumen of an initial segment tubule by in vivo electroporation [84]. Because the spermatozoa in the cauda epididymis are routinely used for IVF experiments, it is possible that the spermatozoa in vivo electroporated with genome editing-related components has a high likelihood of contributing to the generation of offspring with mutated alleles at the target locus via IVF or natural mating.

## Conclusion

*Old methods*: As described above, there are several tested and reported methods for gene delivery to germ cells, all of which have been explored as possible alternatives to PI-based transgenesis. While none of these have been used as routine approaches for producing Tg animals, PI-based transgenesis is by far the most promising and reliable method: it has been thoroughly tested, is reproducible, and is practiced in hundreds of labs worldwide. The PI method, however, requires expensive micromanipulator systems, special skills in the technicians that operate the equipment, ex vivo handling of isolated eggs, and surgical transfer of manipulated eggs to a recipient animal.

*New methods*: Compared to the most commonly used PI method, the recently reported in vitro electroporation-based methods that deliver nucleic acids to pre-implantation embryos, and the *GONAD* method that delivers nucleic acids to embryos in situ in the oviducts, have introduced a new era of generating genome-edited animals in a simplified manner. The *GONAD* system in particular clearly obviates the need for special equipment, isolation of embryos, complex microinjection steps, or microsurgery technicians, all of which are necessary for the PI-based methods.

*Old methods revisited*: Numerous non-PI methods which can successfully deliver nucleic acids components, even though they fail to achieve germ line transmission, could breathe new life into this area of research because of newer genome editing tools that do not require integration into the genome. In our literature search, we found as many as 15 different routes of delivering nucleic acids to eggs, sperm, gonadal tissues, embryos, and fetuses (outlined in Figs. 2, 3 and 4). Nearly half or more of these routes seem to be easily adapted to genome editing using the newer techniques, particularly the CRISPR/Cas9 system, since they need be at the genomic site only for a brief period and do not need to be integrated into the genome. Finally, the CRISPR system can accomplish precise genome editing without leaving any foot-prints on the genome. The CRISPR system is rapidly evolving by the constant addition of new and improved nucleases to its toolbox [85–88]; by using such tools and by revisiting the various delivery approaches discussed in this review, animal genome-editing technology can become even more versatile and efficient in the years to come.

## Point-by-point responses to review comments

### Eugene Koonin (Reviewer 1)

Reviewer Recommendation Term: **Endorse publication**

Please provide a brief overview of your review, stating plainly your opinion of the manuscript’s overall validity, significance and originality.

**Reviewer Comment:** A useful, brief review of nucleic acid delivery methods for genome editing. Obviously, very timely.

Reviewer recommendations to authors

Please make your report as constructive as possible, if necessary, recommending specific improvements so that the authors have the opportunity to overcome any serious deficiencies that you find. Please divide your comments into major and minor recommendations.

**Reviewer Comment:** I think it will be useful to cite these new papers on CRISPR-based tools for genome editing: Zetsche B, Gootenberg JS, Abudayyeh OO, Slaymaker IM, Makarova KS, Essletzbichler P, Volz SE, Joung J, van der Oost J, Regev A, Koonin EV, Zhang F. Cpf1 Is a Single RNA-Guided Endonuclease of a Class 2 CRISPR-Cas System. Cell. 2015 Oct 22;163(3):759–71 Ian M. Slaymaker, Linyi Gao, Bernd Zetsche, David A. Scott, Winston X. Yan, and Feng Zhang. Rationally Engineered Cas9 Nucleases with Improved Specificity. Science, November 2015 DOI: 10.1126/science.aad5227

**Authors’ response:** The papers suggested by the reviewer are now cited, including two more related papers (Shmakov et al. 2015; Kleinstiver et al. 2016). The sentence in the manuscript corresponding to this addition is….

*Also, the CRISPR system can accomplish precise genome editing without leaving any foot-prints on the genome. The CRISPR system is rapidly evolving by constant addition of new and improved nucleases in to its toolbox: by using such tools and by revisiting various delivery approaches discussed in this review, the animal genome editing technology can become even more versatile and efficient in the years to come.*

Minor issues

Please detail any minor comments for the authors attention (spelling, typographical errors, grammatical errors, stylistic suggestions etc.) so that, once addressed, the authors may remove them from the review.

**Reviewer Comment:** no issues

### Haruhiko Siomi (Reviewer 2)

Reviewer Recommendation Term: **Endorse publication**

Custom Review Question(s) Response

Quality of written English

Please indicate the quality of language in the manuscript:

**Reviewer Comment:** Needs some language corrections before being published

Reviewer summary

Please provide a brief overview of your review, stating plainly your opinion of the manuscript’s overall validity, significance and originality.

**Reviewer Comment:** This review summarizes the current state of our knowledge about nucleic acids delivery methods with their history. The review is a very useful recap of the state of the field.

Reviewer recommendations to authors

Please make your report as constructive as possible, if necessary, recommending specific improvements so that the authors have the opportunity to overcome any serious deficiencies that you find. Please divide your comments into major and minor recommendations.

**Reviewer Comment:** I have only a few suggestions to improve its readability. 1. Fig. 1 is not much of help for the reader. The authors shall consider redrawing the cartoon with much fewer words/letters. 2. SMGT is notorious for its no-reproducibility. Do the authors really need to feature this method in their review?

**Authors’ response:**

1. We agree that the Fig. 1 contains many words and letters. We minimized the words in the revised figure.
2. Because this figure gives a concise summary of all the *delivery methods* discussed in the review article, we feel it would be useful to include it in the review.
3. We agree with the reviewer comment about reproducibility issues of SMGT. However, we feel that it is better to include this in the review because of the following reasons: a) this review discusses all previously attempted delivery methods, of which a few of methods (including SMGT) have reproducibility issues… omitting only the SMGT from this review would be inappropriate and b) in the light of CRISPR/cas9 technology, we feel that many of the methods that showed limited success in the past can be re-visited using CRISPR system now, and therefore discussion of all approaches (including poorly reproducible methods such as SMGT) in this review will give the reader a comprehensive list of all the previously attempted delivery methods.

Minor issues

Please detail any minor comments for the authors attention (spelling, typographical errors, grammatical errors, stylistic suggestions etc.) so that, once addressed, the authors may remove them from the review.

**Reviewer Comment:** There are some typographical errors. The authors need careful editing of the manuscript.

**Authors’ response:** The typographical errors are rectified and the manuscript was edited by a professional English Editor. If the Journal still finds that ‘the language needs further improvement’ we will be happy to get it edited by the Journal suggested editorial services

## Abbreviations

ZFN: zinc finger nucleases
TALEN: transcription activator like effector
CRISPR/Cas: clustered regulatory-interspaced short palindromic repeats/CRISPR-associated
HR: homologous recombination
ES cells: embryonic stem cells
DSB: double-strand breaks
NHEJ: non-homologous end joining
HDR: homology-directed repair
PI: pronuclear injection
GOI: gene of interest
KO: knock-out
KI: knock-in
ZP: zona pellucida
SMGT: sperm-mediated gene transfer
ICSI: intracytoplasmic sperm injection
ICSI-MGT: intracytoplasmic sperm injection-mediated gene transfer
GTOVE: gene transfer to oviductal epithelium
GONAD: genome-editing via oviductal nucleic acids delivery
EGFP: enhanced green fluorescent protein
TMGT: testis-mediated gene transfer
Tg: transgenic
IVF: in vitro fertilization
shRNA: short hairpin RNA
sg RNA: single-guide RNA
ST: seminiferous tubule
PI: pronuclear injection
AAV: adeno associated virus
